# Financial Implications of Tariffs for Medical Oxygen on Rwandan Public Hospitals’ Finance Management During the Coronavirus Epidemic

**DOI:** 10.9745/GHSP-D-22-00058

**Published:** 2022-10-31

**Authors:** Diana Kizza, Hyacinth Mushumbamwiza, Siyabonga Ndwandwe, Moyo Butholenkosi, Regis Hitimana, Damien Kirchoffer, Jason Houdek, Eoghan Brady, Logan Brenzel, Nathalie Umutoni, Donatien Bajyanama, Zuberi Muvunyi

**Affiliations:** a Clinton Health Access Initiative, Kigali, Rwanda.; b UNICEF Eastern and Southern Africa Regional Office, Nairobi, Kenya.; c Department of Primary Care and Population Health, University College London, London, United Kingdom.; d Rwanda Social Security Board, Kigali, Rwanda.; e Clinton Health Access Initiative, Barcelona, Spain.; f Clinton Health Access Initiative, Boston, MA, USA.; g Clinton Health Access Initiative, Johannesburg, South Africa.; h The Bill & Melinda Gates Foundation, Seattle, WA, USA.; i Ministry of Health, Kigali, Rwanda.

## Abstract

In Rwanda, provider reimbursements for oxygen are based on the duration of patient consumption at a fixed hourly tariff rate. This study sought to assess whether the current insurance tariff in Rwanda was adequate to cover the costs of oxygen used in oxygen therapy and to explore alternative tariff models.

The assessment found that hospitals make a marginal surplus from low volume flow rate patients and incur losses from patients who require high volume flow rates. In high volume nonspecialized hospitals with a large pool of patients consuming medical oxygen, low flow rate usage patients (e.g., neonates) tend to subsidize high flow usage patients (surgery), if the number of patients consuming low flow oxygen is higher than the latter. The study found that the current tariff was sufficient before the exponential surge in demand for high flow usage during the peak of the COVID-19 pandemic. A variable tariff that factors both the duration (hours) and the volume (liters) used during the therapy may require more work but better reflects the cost of consumption in each ward. A case-based payment model provides a standard pricing framework based on the patient’s diagnosis, intervention, and intensity of treatment.

This study highlights the need for a transition from the time-based tariff structure to a case-based or volume-based tariff to incentivize sustainable production and provision (supply) of medical oxygen services at health facilities in Rwanda. Social health insurance reimbursement tariffs for medical oxygen need to reflect both duration and volume of consumption because oxygen therapy varies based on intervention, disease severity, patient age, length of stay, and responsiveness to treatment.

## Abstract (precis)

This study shows how variations in patient consumption of medical oxygen can be used to determine tariffs more accurately and highlights the need for a transition from the time-based tariff structure to a case-based or volume-based tariff to incentivize sustainable production of medical oxygen services at hospitals in Rwanda.

## Teaser

**Key Findings:**

- The current fee-for-service tariff model in Rwanda does not account for variation in the duration and quantity of oxygen consumed based on intervention, disease severity, and other factors.
- Based on the current tariff model and considering variable oxygen flow rates for different patients, most hospital wards in this analysis were operating at a negative profit margin. However, the total hospital margin over all wards was positive because other wards had much higher profit margins from lower average flow rates.
- Using an alternative tariff model that considers duration and volume of oxygen consumed, particularly when responding to higher needs during future emergencies, will allow hospitals to sustainably procure and supply oxygen. Proper pricing is a critical determinant in the optimal production, procurement, and supply of medical oxygen in hospital management.

**Key Implication:**

- Health insurance policy makers and managers can use our recommendations to design more accurate oxygen pricing models that consider both the duration of the therapy and the volume of oxygen used during the therapy. This will minimize the risk of hospitals operating at a loss and reduce the liability to the insurance company for therapies requiring low flow rates.

*Résumé en français à la fin de l'article*.

## BACKGROUND

Medical oxygen therapy is essential in the treatment of respiratory illnesses including severe coronavirus disease (COVID-19), yet supply remains insufficient in many resource-constrained hospitals.^1^ Despite the 2017 addition of medical oxygen to the World Health Organization Model List of Essential Medicines for the management of hypoxemia,^2^ the global COVID-19 pandemic increased the global visibility of its therapeutic value and subsequently the need to prioritize its supply. This visibility has in turn exposed the already existing systemic bottlenecks hospitals faced, particularly in resource-limited countries, in providing sustainable access to medical oxygen therapy.^3^ The scale of oxygen scarcity has been especially significant in resource-limited countries including India^4^ and many countries in sub-Saharan Africa.^5^^,^^6^ The bottlenecks include limited supply-chain capacity,^1^ unreliable electrical power needed to deliver oxygen at the point of care,^5^ inadequate health care provider training at facilities,^7^ and limited capacity and government resources to maintain local oxygen production.^8^^–^^10^

In Rwanda, oxygen production capacity has grown over time due to the increased need associated with the COVID-19 pandemic. Before the pandemic, there were 9 pressure swing adsorption (PSA) oxygen plants in Rwanda—2 privately owned and 7 owned by public hospitals. The plants at public hospitals produce oxygen for internal consumption and sell the surplus to peripheral hospitals. The revenue from these sales contributes to the hospitals’ internally generated revenue, for which hospitals have full autonomy and fiscal responsibility.

By February 2020, national consumption was 6,760 cylinders (50 L, 150 bar) per month, and public health care facilities accounted for 68% of the supply. Public plants collectively operated only at 64% capacity, producing 156 cylinders (50 L, 150 bar) of oxygen per day versus 242 cylinders (50 L, 150 bar) per day capacity. Only 5 of the 7 public plants operated at full capacity. Both supply and demand factors contributed to this suboptimal performance. Meanwhile, delayed payments from surplus sales, irregular plant maintenance, and reportedly low reimbursement tariffs—which providers argued did not significantly cover costs—disincentivized production, ultimately affecting supply. For example, oxygen consumption data from the University Teaching Hospital of Kigali and Ruhengeri Hospital show that consumption represents only 48% of the expected clinical need for oxygen (based on the per bed by ward and overall bed occupancy rates). This ratio varies by province—the Eastern and Western provinces consume the least oxygen and have the least accessibility to production hubs. In addition, clinicians were underdiagnosing hypoxemia due to a lack of adequate oximeters resulting in low and irregular orders.

During the initial response to the pandemic, production could not be increased immediately, leading to supply gaps, and public hospitals with plants prioritized providing oxygen to COVID-19 designated treatment centers, following demand patterns. Since February 2020, additional PSA plants have been installed in response to the pandemic, primarily at COVID-19 treatment centers to manage and control severe COVID-19 cases. To date, 22 new PSA plants have been added, increasing the number of PSA plants located in public health care facilities to 29.

Health facilities in Rwanda purchase oxygen by volume. The average procurement cost price of oxygen is Rwandan Francs [RWF]15000 (US$16) per cylinder (300 L). Insurers, in contrast, have a single hourly medical oxygen therapy tariff and reimburse hospitals based on the number of hours that beneficiaries spend on oxygen therapy regardless of volume consumed. This reimbursement structure poses problems because the Rwanda Clinical Guidelines for Hypoxemia Screening and Oxygen Therapy Administration in Neonates, Children and Adults recommend different oxygen flow rates (i.e., volumes) to treat various medical conditions.

However, it was uncertain whether the current tariff structure is sufficient to cover the costs incurred by facilities in purchasing and producing oxygen in the long term, especially during periods of increased demand for high-volume therapy. Health care facilities' ability to meet their costs of production influence their ability to continue to produce oxygen sufficiently. In Rwanda, human resources, medical accessories, and fixed costs such as oxygen distribution piping installation across essential wards, maintenance, and repairs are covered by the government’s global budgets from the Ministry of Finance and Economic Development. Variable costs including oxygen are reimbursed by health insurers; the dominant insurer is the Community Based Health Insurance (CBHI), which in 2021 covered 85.6% of the population.^11^ The CBHI scheme also reimburses 85% of facility oxygen therapy utilization (in hours) and has the lowest tariff (Table 1).

**TABLE 1.** Tariffs for Medical Oxygen by Financing Sources, Rwanda

**Insurance Schemes**	**Tariff, RWF (US$)** ^a^	**Total Amount Reimbursed, %**
Rwanda Social Security Board-Community Based Health Insurance^b^	635 (0.67)	85.1
Rwanda Social Security Board-Rwandaise d'Assurance Maldie^b^	1125 (1.20)	2.5
Other insurance	1035 (1.11)	4.8
None^c^	1656 (1.77)	7.5

Abbreviation: RWF, Rwandan franc.

^a^ US$1=RWF935.

^b^ Rwanda Social Security Board includes 2 types of insurance schemes. Rwandaise d'Assurance Maldie provides civil servants with additional benefits and simpler administration, and the mandatory Community Based Health Insurance Scheme covers the rest of the population.

^c^ Refers to patients with no insurance or no recognized insurance.

It was uncertain whether the current tariff structure is sufficient to cover the costs incurred by facilities in purchasing and producing oxygen in the long term, especially during periods of increased demand for high volume therapy.

To the best of our knowledge, there has not been a formal assessment of whether the current arrangements for the production and consumption of medical oxygen meet the demand and supply of medical oxygen for health facilities in the long term. In this study, we assessed whether the current tariff rates allow hospitals to break even by comparing the variable oxygen procurement costs against CBHI reimbursement for hospital wards that used medical oxygen therapy. This is an important consideration, as it impacts how much medical oxygen can be produced, procured, and ultimately whether all patients in need of oxygen therapy can have equitable and sustainable access to the therapy they need. We also describe the current financing structures for oxygen in Rwandan health facilities and explore different tariff models that may be fairer for both the health provider and the payer.

## METHODS

Rwanda currently operates a well-functioning, decentralized health care public service system comprising 1,060 health posts, 500 health centers, 42 district and provincial hospitals, and 5 national referral hospitals. Rwanda also has a private health services sector that comprises 2 general hospitals, 2 eye hospitals, 50 clinics and polyclinics, 8 dental clinics, 4 eye clinics, and 134 dispensaries.^8^ Public hospitals make up the larger proportion (64%) of facilities, and utilization is 1.8 visits per patient annually. We compared the oxygen procurement cost and insurance claims for 25 hospitals—20 district hospitals, 4 referral hospitals, and 1 provincial hospital—between June 2019 and June 2020. Importantly, most of the study period occurred before the onset of the COVID-19 pandemic in Rwanda.

In assessing whether the current fixed tariff adequately covers procurement costs of medical oxygen by the facilities, and under what circumstances would a variable tariff help hospitals break even, we conducted 2 assessments. First, we estimate the financial implications of the current fixed tariff that only factors the duration (hours) of oxygen consumed to hospitals at the individual ward level. Second, we estimated a variable tariff that factors both the duration (hours) and the volume (liters) that closely links the amount of oxygen consumed and the variable cost of providing the therapy.

To estimate the clinical oxygen therapy utilization of patients, we collected administrative data on admissions, diagnosis, treatments, therapy flow rates, and treatment duration from electronic hospital management records^12^ for 37,809 patients in the 25 hospitals between June 2019 and June 2020, across an average of 9 wards per hospital including neonatology, internal medicine, pediatric, emergency, surgery, anesthesia, maternity, and outpatient department. Consumption per patient was estimated using the average recommended oxygen consumption per ward, as stipulated in the national clinical guidelines (Table 2). The Clinton Health Access Initiative’s good data handling practice procedure was adopted for all patient-identifiable information in receiving, transiting, and analyzing data, as well as upon completion of data analysis.

**TABLE 2.** Clinical Guidelines for Oxygen Therapy in Rwanda

Hospital Wards	Average Flow Rate, LPM
Intensive care unit	10
COVID-19 isolation	10
Neonatology	1
Internal medicine	6
Pediatrics	2
Obstetrics and gynecology	6
Minor surgery and post operations	6
Emergency	10
General	6

Abbreviations: COVID-19, coronavirus disease; LPM, liters per minute.

We estimated the financial costs incurred by hospitals based on their revenue from insurance reimbursement claims and the total oxygen procurement costs. The recurring procurement costs (oxygen cylinder refills and transport costs only, excluding the gas cylinder cost) and reimbursement revenues of oxygen were obtained from the finance departments of the same hospitals based on the unit sales price per cylinder and the reimbursements received from insurers.

The hospital revenue and margins from oxygen therapy were estimated based on the reported hours of treatment, average flow rates from 2012 clinical guidelines, the tariffs from different insurance schemes and noninsured patients, and the average price of oxygen procurement. The sufficiency and financial implications of the tariffs for hospitals were estimated based on the margins between revenue data and total oxygen procurement costs.

In Rwanda, oxygen is reimbursed at a fixed rate determined by time per hour at RWF635 (US$0.68) per hour for a patient on the CBHI scheme) and procured in volumes per liter from the supplier at an average of RWF300 (US$0.32) per liter of compressed gas oxygen. Since the pandemic resulted in a surge in volume of high flow oxygen therapy, we created an alternative tariff model to compare against the existing tariff model. An alternative model would consider both volume and time to address the variances in usage across wards (Box).

BOX **Equations Used to Model Alternative Tariff Reimbursement Rate for Oxygen in Rwanda Health Facilities**

To model this alternative tariff, we considered the following:

1. The flow rate (liters per minute [LPM]) is proportional to the severity (S) of the illness and the condition (C) being managed,

$$\frac{\delta L}{\delta t}=kSC$$

where $\frac{\delta c}{\delta L}$ is the rate per liter of oxygen consumed.

2. The tariff therefore is a function of both the duration (hours) and the volume (liters) used during the therapy,

$$A=kt+\frac{\delta c}{\delta L}L$$

Where A is the alternative tariff, t is time in hours, L is the volume of oxygen in liters, k is the cost of oxygen per hour of consumption, and $\frac{\delta c}{\delta L}$ is the rate per liter of oxygen consumed.

3. Solving this equation gives the following result

A=198.5t+2L

For comparison to the existing tariff model, we created an alternative model that would consider both volume and time to address variances in oxygen usage across wards.

Studies show that approximately 10%–15% of the patients with COVID-19 will require oxygen therapy.^13^^–^^15^ As of September 24, 2021, the Rwanda Biomedical Center reported the cumulative total of positive COVID-19 cases in Rwanda at 96,337. Extrapolating from the experience in India, for inference, we implied that between 9,634 and 14,451 cases in Rwanda would have required oxygen therapy in this period.

Survival analysis findings established that the median time to complete supplemental oxygen therapy among the studied population for COVID-19 patients was 6 days (144 hours).^16^ While oxygen therapy is recommended for the management of COVID-19 patients, the methods of administration vary according to severity. The oxygen flow rates typically vary from 2 to 15 LPM.^17^ Therefore, we assessed the potential revenue based on use of the variable tariff rate in comparison to the fixed tariff rate within the oxygen flow rate range of 2 to 15 LPM.

### Limitations of the Methodology

Due to limited availability and data aggregation, the analysis did not use actual oxygen volume consumed but estimated volumes based on clinical guidelines and reported duration of treatment. It is likely that actual clinician practices deviate from the norms due to the clinician’s low adherence to guidelines and oxygen shortages. We have no way of knowing the actual volume used per patient as these data are not recorded. This analysis does not consider the clinical inaccuracy of estimated oxygen uptake (resting using Fick’s equation nor measured using Dehmer's formula) but rather the financial perspective that influences oxygen procurement by the cylinder. The study also does not delve into the parameter of oxygen leakage in the administration of oxygen therapy, nor the reimbursement against chargeable weight in road transportation of oxygen cylinders. In the context of COVID-19 case management, a general limitation of the study is that the analysis does not consider the effect of comorbidities, auxiliary management for COVID-19, and oxygen procurement when there are other sources of oxygen as liquid oxygen.

## RESULTS

We aimed to answer 2 questions about the tariff rate on whether the current fixed tariff adequately covers procurement costs of medical oxygen by the facilities and under what circumstances would a variable tariff help hospitals break even.

### Is the Current Fixed Tariff Adequate To Cover Hospitals’ Oxygen Procurement Costs?

#### Usage

**FIGURE 1:**
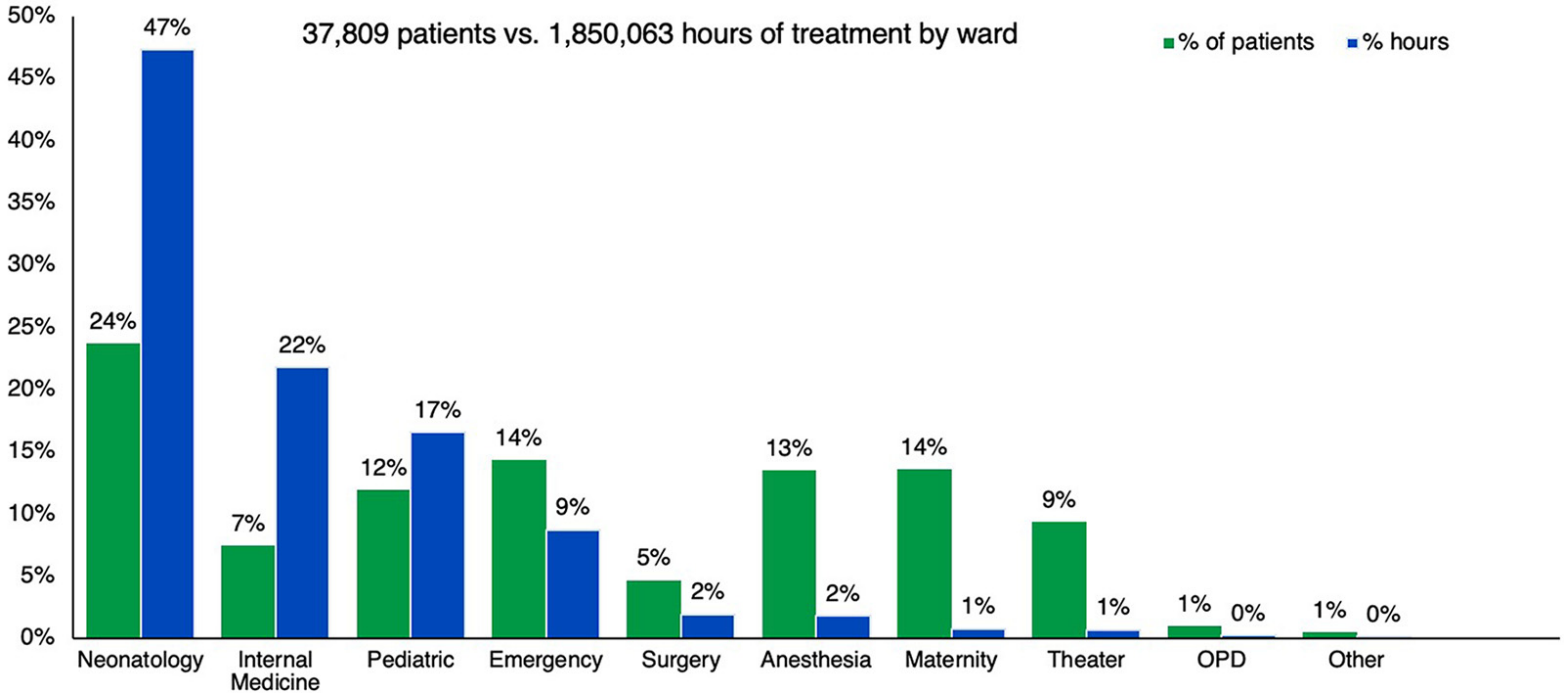
Comparison of Patients and Total Oxygen Consumption by Hospital Ward, Rwanda, June 2019 to June 2020 Abbreviation: OPD, outpatient department.

**FIGURE 2:**
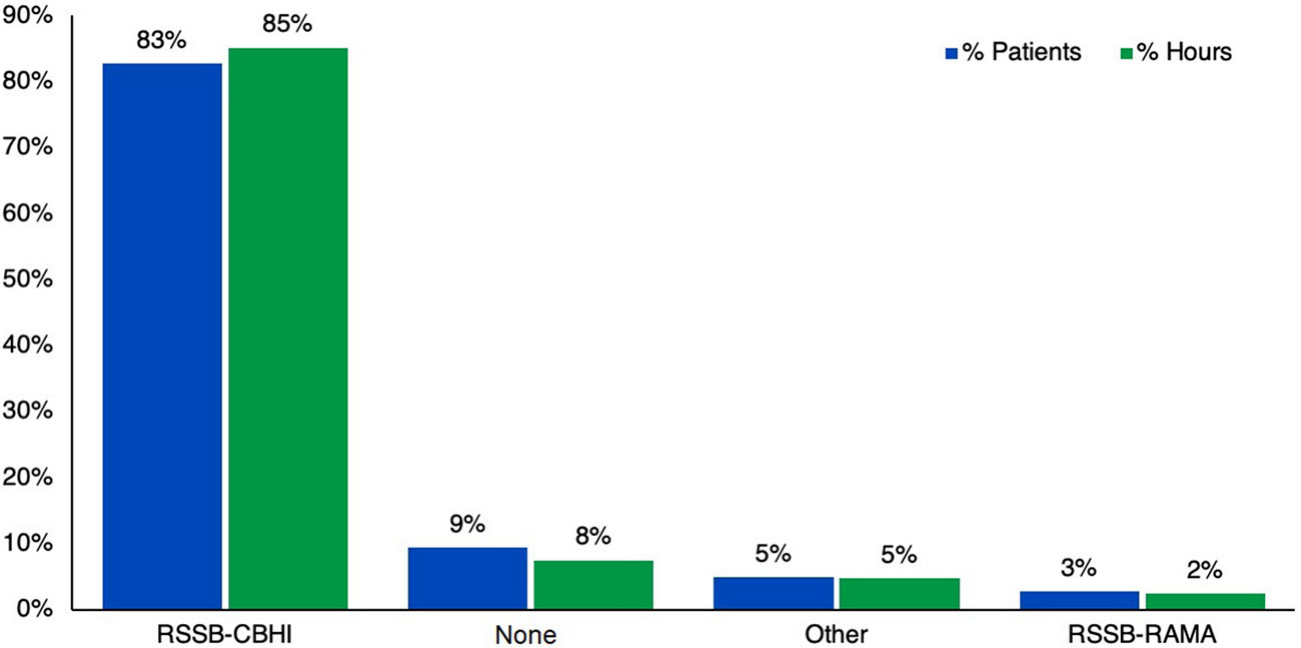
Patient Source of Financing for Oxygen Therapy in Rwanda Abbreviations: RSSB-CBHI, Rwanda Social Security Board-Community Based Health Insurance; RSSB-RAMA, Rwanda Social Security Board-Rwandaise d'Assurance Maldie.

**FIGURE 3:**
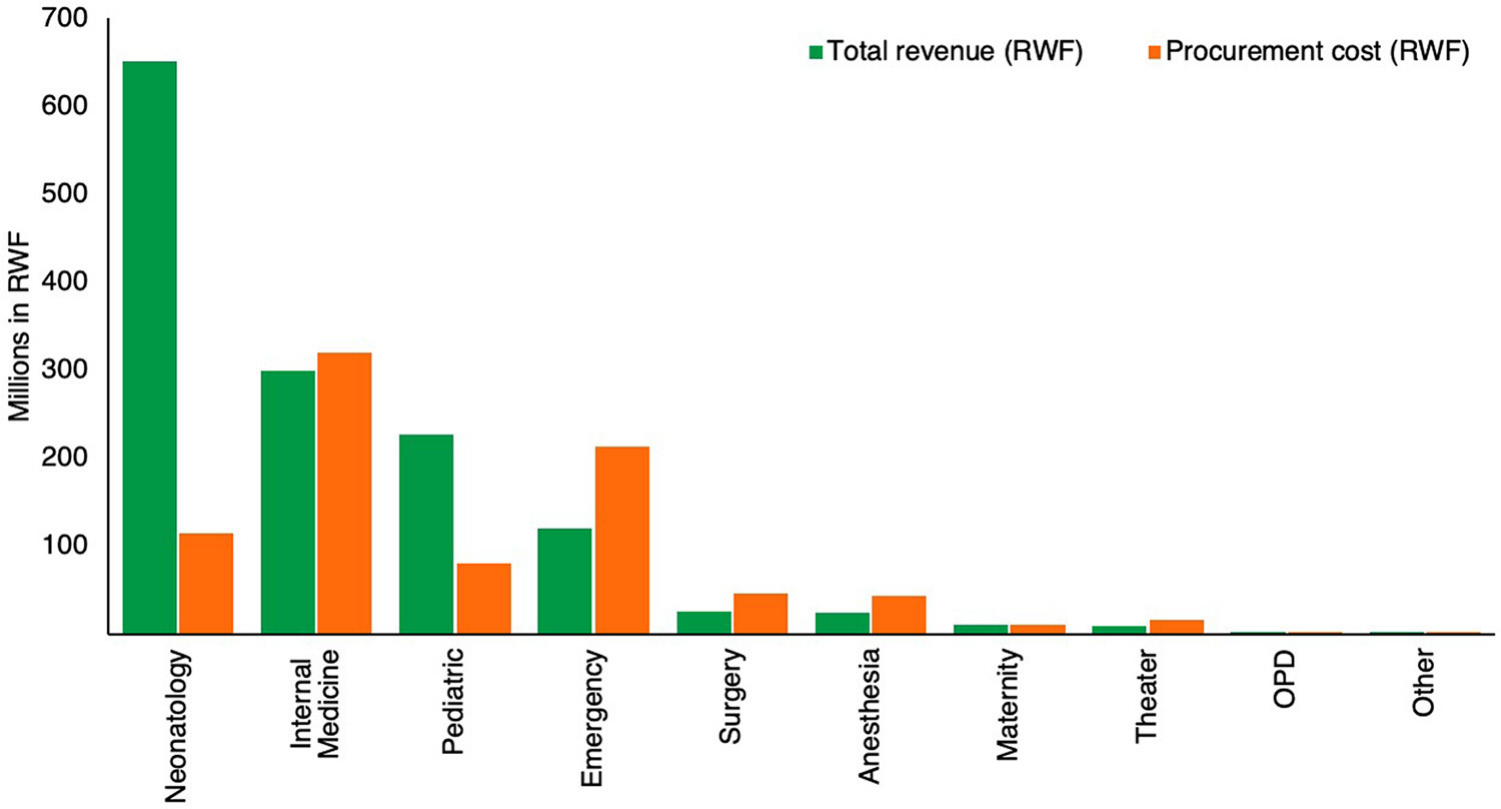
Hospital Revenue Versus Oxygen Procurement Costs in Rwanda Abbreviations: OPD, outpatient department; RWF, Rwandan franc.

Of the 37,809 total patients who received oxygen during the study period, most patients who received oxygen therapy were from the neonatology ward (24%) followed by 14% from the emergency ward, while OPD accounted for one of the lowest number of patients (1%) (Figure 1). In terms of duration of treatment, neonatology and pediatric wards combined, which represent the low-volume consumption interventions, represent 64% of the total patients. Forty-seven percent of the total oxygen therapy hours were for neonates followed by 22% for patients in internal medicine, with outpatient department patients using the lowest percentage of hours (0.2%). Figure 2 shows that CBHI, which offers the lowest tariff, accounted for 85% of the oxygen therapy hours. In terms of quantity of oxygen consumed, Figure 3 shows that while internal medicine and emergency combined used about 62% of the total oxygen, 64% of the total revenue was collected from patients in the neonatology and pediatrics departments.

While internal medicine and emergency combined used about 62% of the total oxygen, 64% of the total revenue was collected from patients in the neonatology and pediatrics departments.

#### Cost, Revenue, and Profitability

At the facility level, the current tariff structure can allow hospitals to break even; however, some departments incur significant losses that end up being subsidized by surpluses made by other departments. The mean oxygen flow rate, rather than the number of patients, highly reflects whether a department makes a profit or loss from providing oxygen therapy (Table 3).

**TABLE 3.** Profit Margins Using Mean Flow Rates, by Hospital Ward, Rwanda

**Department**	**Share of Patients Requiring Oxygen, %**	**Share of Hours of Oxygen Use, %**	**Mean Flow Rates, Liters/Minute**	**Profitability/ Margins, %**
Emergency	14	9	10	−44
Surgery	5	2	10	−44
Anesthesia	13	2	10	−44
Theater	9	1	10	−44
Internal medicine	7	22	6	−6
Maternity	14	1	6	−6
Outpatient department	1	0	6	−6
Other	1	0	6	−6
Pediatric	12	17	2	181
Neonatology	24	47	1	462

**FIGURE 4:**
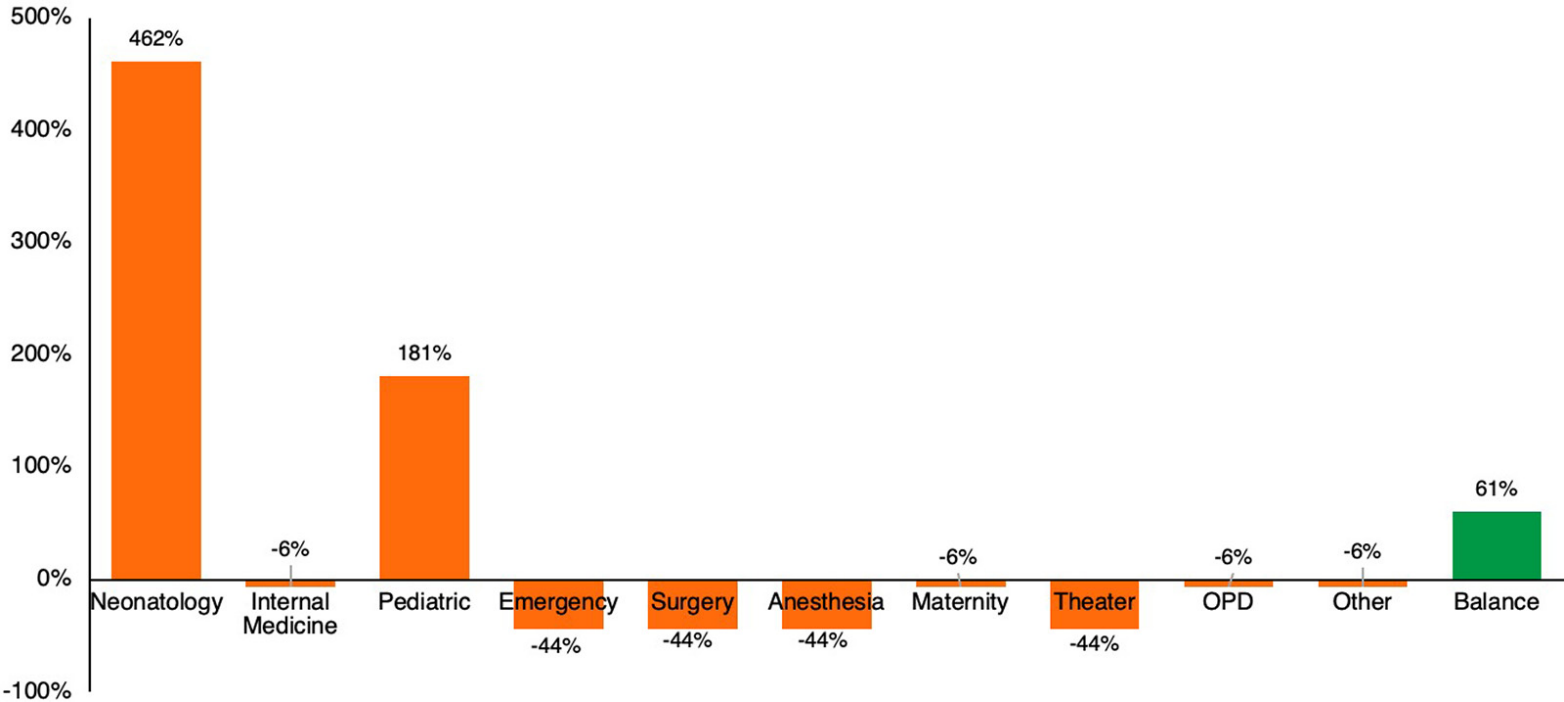
Profit Margins From Oxygen Costs by Ward in Rwanda Abbreviation: OPD, outpatient department.

The margin on oxygen therapy most departments generate is negative (Figure 4). However, the total hospital margin reaches 61% on average of the total procurement cost due to positive margins from neonatology and pediatrics patients. As previously mentioned, this margin is explained by the low average flow rates administered to neonates and pediatrics patients relative to other departments.

Overall, the analysis shows that the current flat hourly tariff is adequate to cover hospital costs, given the cumulative oxygen consumption across all the wards. Hospitals are making sufficient margins from the oxygen tariffs, including CBHI, to cover procurement costs. However, this conclusion does not apply to settings where a substantial number of patients requiring high oxygen flow rates need treatment.

The current flat hourly tariff is adequate to cover hospital costs given the oxygen use across all wards.

### In What Circumstances Would a Variable Tariff Be More Adequate?

The COVID-19 pandemic has drastically increased the quantity of oxygen demanded. The second outbreak between January 2021 and March 2021 in Kigali highlighted the drastic need for oxygen and propelled immediate construction of a new PSA plant at the main treatment center in Nyarugenge to meet that demand. Because the COVID-19 pandemic changed the assumptions on cumulative oxygen consumption in a hospital, we modeled an alternative tariff that factors both oxygen therapy duration and oxygen volume used.

**FIGURE 5:**
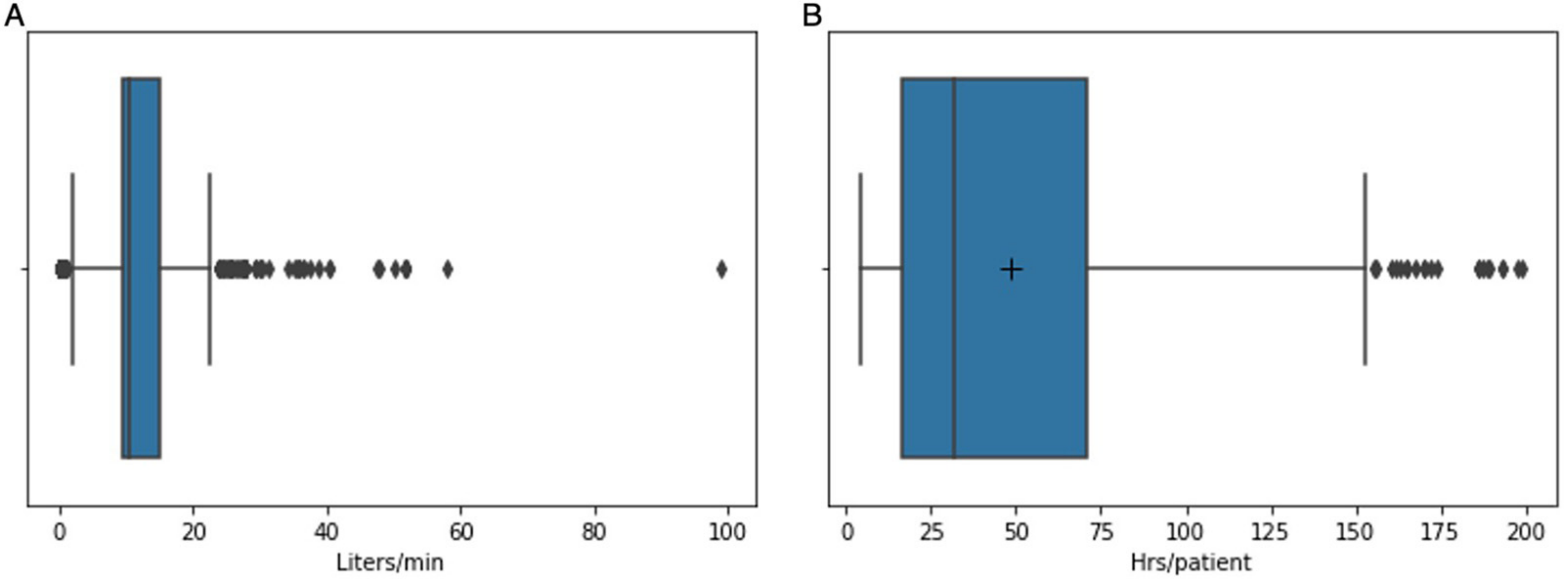
Box Plot of Oxygen Use During Patient Treatment (A) Distribution of the Rate Used During Treatment and (B) Distribution of the Duration of Oxygen Treatment^a^ Abbreviations: hrs, hours; min, minute. ^a^ The box represents the lower and upper quartiles of the middle 50% of the data. The line in the box represents the median of that data. The ends of the whiskers represent the nonusual minimum and maximum. Dots represent outliers, and extreme dots represent minimum and maximum values. The plus sign represents the mean duration, which when contrasted with the median, exposes the skewness of the distribution.

**FIGURE 6:**
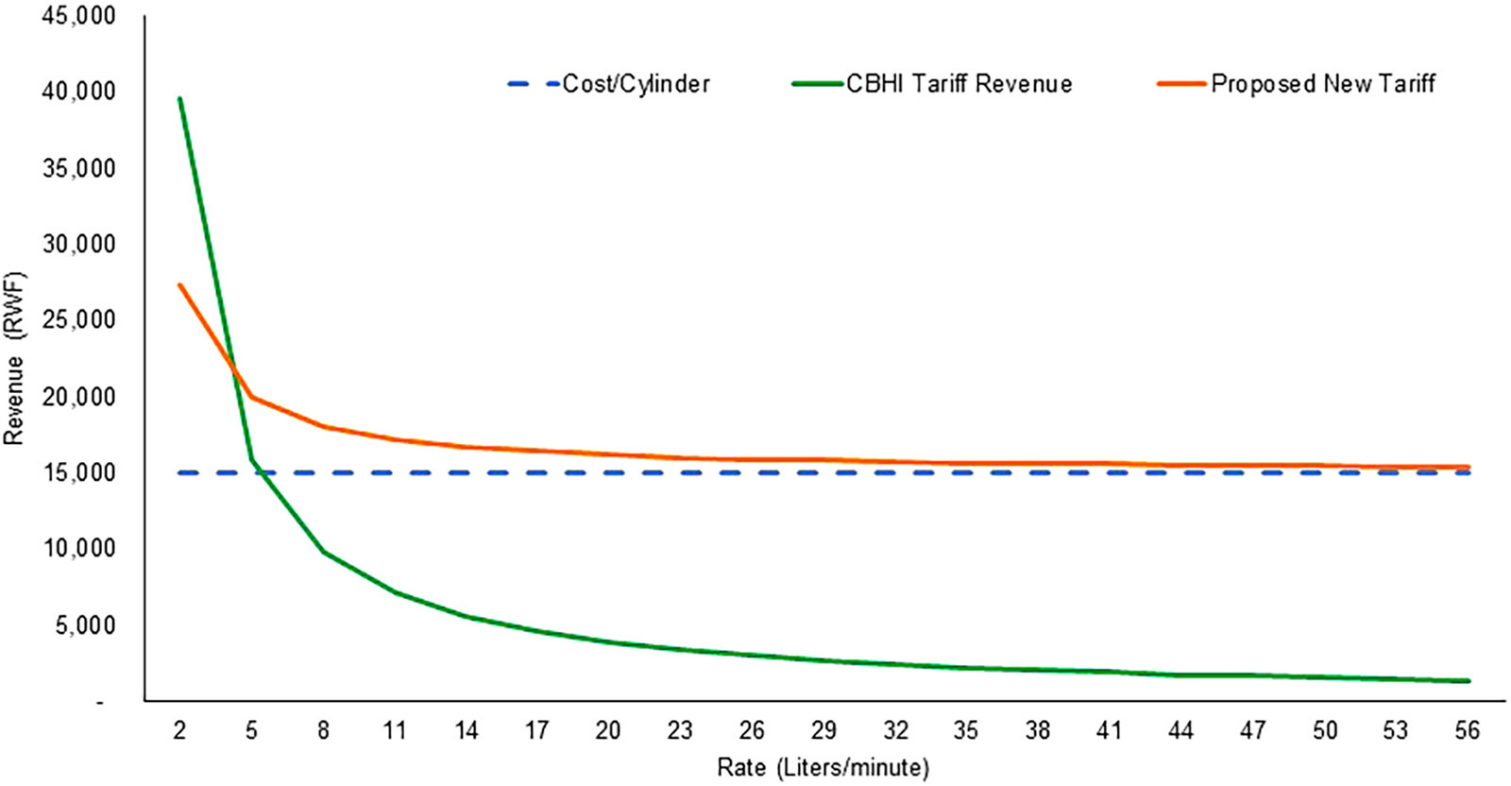
Oxygen Revenues Based on An Alternative Insurance Reimbursement Tariff Model in Rwanda Abbreviations: CBHI, Community Based Health Insurance; RWF, Rwandan franc.

From the electronic medical records and claims data for 37,809 non-COVID-19 patients between June 2019 and June 2020, we determined the median flow rate was approximately 10 LPM (with the upper quartile having a wider variation of flow rates) and the median duration of oxygen therapy was 50 hours per patient (Figure 5). Evidence shows that during treatment for COVID-19, oxygen therapy increases in duration to between 68 and 75 hours per patient.^16^ Considering that a flow rate higher than 4 LPM on non-COVID-19 patients covered by CBHI pushes service providers into a loss (Figure 6), this duration of high flow oxygen therapy would greatly increase the loss to providers. However, under the current oxygen therapy tariffs and current oxygen consumption, hospitals generate an average margin of 69% above their oxygen procurement cost, which is about RWF180 (US$0.19) per liter. The alternative variable tariff that considers both the duration of the therapy and the volume of oxygen used during the therapy is estimated to minimize the marginal revenue obtained at lower flow rates while also minimizing the loss obtained while using high flow rates.

## DISCUSSION

Health insurance arrangements were instrumental in containing the pricing of medical oxygen during the surge in demand caused by the COVID-19 pandemic, where prices shot up more than sevenfold times and hoarding was observed in unregulated markets.^18^ However, this brought to the fore the health facility concerns around fair pricing of a fixed hourly insurance tariff rate amidst high volume consumption at high volume flow rates. The limitation of the reimbursement tariff rate is that prescribed oxygen therapy flow rates vary between medical conditions, yet only 1 rate is charged irrespective of the volume used. In addition, procurement is computed in volume per cylinder procured, which is a different unit from that used to determine the reimbursement tariff. Social health insurance reimbursement tariffs for medical oxygen need to reflect both duration and volume of consumption because oxygen therapy varies based on intervention, disease severity, patient age, length of stay, and responsiveness to treatment. As a best practice for price setting, prices should reflect the cost of delivering services efficiently to minimize incentives for inappropriate or low-value care and to enable hospitals to make accurate budget projections.^19^

Under normal utilization, hospitals in Rwanda yield marginal revenue surplus from low volume flow rate patients and incur losses from patients who require high volume flow rates. In high volume nonspecialized hospitals with a large pool of patients consuming medical oxygen, low flow rate usage patients (e.g., neonates) tend to subsidize high flow usage patients (e.g., in surgery) only if the number of patients consuming low flow oxygen is higher. This study found that the tariff was sufficient before the exponential surge in demand for high flow usage during the peak of the COVID-19 pandemic and intensive care unit expansions. This fixed hourly tariff model has implications on affordability for highly specialized facilities and hospitals during unprecedented demand surges.

Based on the results, this study explored 2 models for computing oxygen tariffs that countries can compare in determining the most optimal reimbursement rate.

1. A fixed tariff that only factors in the duration (hours) of oxygen consumed requires proper data capture by the clinical staff on the hours consumed per patient. It is assumed that high flow rate wards will be subsidized by low flow rate wards, and as such the tariff will be sufficient as observed with the data from Rwanda. However, if a facility has a high volume of intensive care unit patients in comparison to neonates and maternal patients, then this model will not result in optimal financial management for the hospital. In Rwanda, the bulk of oxygen is produced at hospital-managed PSA plants. Such a tariff may not incentivize a competitive private sector unless a public-private partnership is designed.

2. A variable tariff that factors both the duration (hours) and the volume (liters) used during the therapy may require more work but better reflects the cost of consumption in each ward. This model almost always guarantees that the hospitals will not operate at a loss while also reducing the liability to the insurance company for therapies requiring low flow rates. This model is more difficult to implement due to the administrative costs of measuring and reimbursing on this basis.

A variable tariff model factoring both duration and hours almost always guarantees that hospitals will not operate at a loss while also reducing liability to the insurance company for therapies requiring low flow rates.

3. A third policy recommendation would require a shift from traditional fee-for-service or line budgets to case-based payment models. With this model, instead of paying for oxygen as a specific service provided, the tariff is predetermined based on a diagnostic-related group. According to the European Observatory on Health Systems and Policies glossary, case-based payments are where insurers pay physicians/hospitals according to the workload of cases treated rather than per service or bed days, either based on a single flat rate per case or on a schedule of payment by diagnosis, often based on diagnostic-related groups. This adopts a standard pricing framework that provides a technical means for the management and financing of public hospital services, linked with social health insurance and government funding mechanisms. While this shift takes time, it has been widely accepted as necessary to reduce the per capita cost of health care across the Asia Pacific region.^20^

Overall, this study informs policy makers on the link between prices and supply of oxygen therapy through an understanding of how variations in patient consumption can be used to accurately determine tariffs. This scenario mirrors the challenges in setting optimal tariffs and pricing for other diagnostics, medical consultations, and the proper quantification of drugs for the reimbursements of public providers, particularly in systems that use traditional fee-for-service payment mechanisms. Similar analyses should be conducted for other high-volume drugs and diagnostic services for which insurance reimbursement rates are often challenged by providers and suppliers.

### Scope for Future Studies

Future studies in Africa should examine how adequate pricing is determined for other health services and how insurance tariffs affect incentives for sufficient service delivery. Also, a study on oxygen wastage from clinical inefficiencies due to limited use of pulse oximeters and poor equipment maintenance would help to further refine pricing methods.

### Limitations

Oxygen wastage was not factored in the study. While oxygen wastage is difficult to capture, improved clinical administration with a more consistent adaptation of the use of pulse oximeters can help regulate the oxygen flow as patients stabilize—practices that need to be improved to control effective utilization and costs. Given the complexities of measuring and billing oxygen based on consumption, and the likelihood that wastages might also be invoiced and reimbursed as consumed by patients—which does not provide enough incentive to minimize wastages—we recommend moving away from fee-for-service payment to other payment methods such as case-based payment or diagnostic-related groups but with good cost estimations for oxygen based on duration and volume.

Additional cost barriers that limit optimal oxygen supply were not factored into this tariff discussion. Hospitals are not always able to manage and plan PSA production resources to factor in maintenance costs for PSA plants. When the plants break down due to irregular maintenance, supply is cut for extended periods until supplementary budgets are secured from the government. Historically, private contractors were awarded maintenance contracts for PSA plants, but during the period of the study, their contracts had yet to be renewed. Hospitals have autonomy in choosing PSA plant brands. While this promotes competition, given the total number of plants, it also leads to inefficiencies in maintenance as spares are sought from multiple vendors.

Lastly, capacity gaps in biomedical maintenance teams and clinical practice on oxygen therapy due to the limited use of protocols and standards of procedures also lead to suboptimal consumption, particularly where there is limited pulse oximetry monitoring of patients on oxygen therapy to guide a gradual reduction in oxygen therapy as patient recover. Originally, hospitals were subjected to noncommercial electricity costs that also drove up the costs of oxygen production plants and concentrators. Hospitals in Rwanda were provided subsidized electricity rates in 2019 when this concern was presented to the Rwanda Electricity Authority. These additional financial and economic costs were not considered in this study.

## CONCLUSION

Social health insurance reimbursement tariffs for medical oxygen need to reflect both duration and volume of consumption because oxygen therapy varies based on intervention, disease severity, patient age, length of stay, and responsiveness to treatment. A single oxygen tariff needs to reflect these multiple parameters so that hospitals can sustainably afford to procure or produce this essential medicine. This will minimize the marginal revenue obtained at lower flow rates while also minimizing the loss obtained while using high flow rates.

## Translation

**En Français**

**IMPLICATIONS FINANCIÈRES DES TARIFS DE L’OXYGÈNE MÉDICAL SUR LA GESTION FINANCIÈRE DES HÔPITAUX PUBLICS RWANDAIS PENDANT L’ÉPIDÉMIE DE CORONAVIRUS**

**Résumé**

Au Rwanda, les remboursements des fournisseurs d’oxygène sont basés sur la durée de consommation des patients à un taux horaire fixe. Cette étude visait à évaluer si le tarif d’assurance actuel au Rwanda était suffisant pour couvrir les coûts de l’oxygène utilisé en oxygénothérapie et à explorer d’autres modèles tarifaires.

L’évaluation a révélé que les hôpitaux génèrent un excédent marginal avec les patients nécessitant de faibles volumes d’oxygène et subissent des pertes avec les patients qui ont besoin de débits de volume élevés. Dans les hôpitaux non spécialisés à volume élevé avec un grand nombre de patients consommant de l’oxygène médical, les patients à faible débit (par exemple, les nouveau-nés) ont tendance à subventionner les patients à haut débit (chirurgie), si le nombre de patients consommant de l’oxygène à faible débit est plus élevé que ce dernier.

L’étude a révélé que le tarif actuel était suffisant avant l’augmentation exponentielle de la demande d’utilisation à haut débit pendant le pic de la pandémie de COVID-19. Un tarif variable qui tient compte à la fois de la durée (heures) et du volume (litres) utilisés pendant la thérapie peut nécessiter plus de travail, mais reflète mieux le coût de la consommation dans chaque service. Un modèle de paiement basé cas par cas fournit un cadre de tarification standard basé sur le diagnostic, l’intervention et l’intensité du traitement du patient.

Cette étude souligne la nécessité d’une transition de la structure tarifaire basée sur le temps à un tarif basé cas par cas ou sur le volume afin d’encourager la production durable et la fourniture (fourniture) de services d’oxygène médical dans les établissements de santé au Rwanda. Les tarifs de remboursement de l’assurance maladie sociale pour l’oxygène médical doivent refléter à la fois la durée et le volume de consommation, car l’oxygénothérapie varie en fonction de l’intervention, de la gravité de la maladie, de l’âge du patient, de la durée du séjour et de la réactivité au traitement.

**Peer Reviewed**

**First published online:** October 4, 2022.
